# HER2 chimeric antigen receptor T cell immunotherapy is an effective treatment for diffuse intrinsic pontine glioma

**DOI:** 10.1093/noajnl/vdad024

**Published:** 2023-05-04

**Authors:** Stacie S Wang, Alexander J Davenport, Melinda Iliopoulos, Hannah E Hughes-Parry, Katherine A Watson, Valeria Arcucci, Matthias Mulazzani, David D Eisenstat, Jordan R Hansford, Ryan S Cross, Misty R Jenkins

**Affiliations:** Immunology Division, The Walter and Eliza Hall Institute of Medical Research, Parkville, VIC, Australia; Murdoch Children’s Research Institute, Parkville, VIC, Australia; Children’s Cancer Centre, Royal Children’s Hospital, Parkville, VIC, Australia; Immunology Division, The Walter and Eliza Hall Institute of Medical Research, Parkville, VIC, Australia; Immunology Division, The Walter and Eliza Hall Institute of Medical Research, Parkville, VIC, Australia; Immunology Division, The Walter and Eliza Hall Institute of Medical Research, Parkville, VIC, Australia; Immunology Division, The Walter and Eliza Hall Institute of Medical Research, Parkville, VIC, Australia; Immunology Division, The Walter and Eliza Hall Institute of Medical Research, Parkville, VIC, Australia; Immunology Division, The Walter and Eliza Hall Institute of Medical Research, Parkville, VIC, Australia; Murdoch Children’s Research Institute, Parkville, VIC, Australia; Children’s Cancer Centre, Royal Children’s Hospital, Parkville, VIC, Australia; Michael Rice Cancer Centre, Women’s and Children’s Hospital, South Australia Health and Medical Research Institute, South Australia ImmunoGENomics Cancer Institute, University of Adelaide, Adelaide, South Australia, Australia; Immunology Division, The Walter and Eliza Hall Institute of Medical Research, Parkville, VIC, Australia; Immunology Division, The Walter and Eliza Hall Institute of Medical Research, Parkville, VIC, Australia; Department of Medical Biology, University of Melbourne, Parkville, Australia; Department of Biochemistry, La Trobe Institute for Molecular Science, Bundoora, VICAustralia

**Keywords:** Chimeric antigen receptor (CAR) T cells, diffuse intrinsic pontine glioma (DIPG), diffuse midline glioma (DMG), human epidermal growth factor receptor 2 (HER2), immunotherapy

## Abstract

**Background:**

Diffuse intrinsic pontine glioma (DIPG) and other diffuse midline gliomas (DMG) of the thalamus and spinal cord are rare but devastating high-grade glial tumors of childhood with no curative treatment. Despite aggressive treatment attempts the prognosis has remained poor. Chimeric antigen receptor (CAR) T cell therapy has been identified as a promising new approach in the treatment of DMG tumors; however, additional targets are urgently required given known tumor heterogeneity and the prospect of antigen escape of this cancer.

**Methods:**

Using cell surface mass spectrometry, we detected high HER2 cell surface protein across a panel of patient-derived DIPG cells, thereby identifying an existing CAR T cell therapy for use in DIPG. Primary human T cells were transduced to express a second-generation HER2 CAR and interrogated for efficacy against patient-derived DIPG cells.

**Results:**

HER2 CAR T cells demonstrated potent and antigen-specific cytotoxicity and cytokine secretion when co-cultured with patient-derived DIPG cells. Furthermore, HER2 CAR T cells provided a significant regression in intracranial DIPG xenograft tumors.

**Conclusions:**

HER2 CAR T cells are already in clinic development and are well tolerated in pediatric patients. Here we provide strong preclinical evidence for the inclusion of DIPG patients in future pediatric CNS tumor HER2 CAR T cell clinical trials.

**Key Points**

- This study shows some DIPG tumors highly express HER2 which can be exploited for CAR T cell immunotherapy.
- HER2 CAR T cell clinical trials, currently recruiting pediatric patients with CNS tumors, should expand to include all DIPG patients.

**Importance of the Study**

DMG, including DIPG, is an almost universally fatal CNS tumors of childhood and is the only tumor indication wherein the current standard of care is palliative radiotherapy. These children typically have a dismal prognosis with a median survival of only 9 months. Previous attempts at new therapies have all failed to meet this urgent unmet clinical need and there is a paucity of new approaches. Chimeric antigen receptor (CAR) T cells are providing the first wave of hope in this devasting disease, but are likely to fail as a monotherapy; therefore multiple targets need to be validated to facilitate a multipronged approach. Here we show that HER2 CAR T cells elicit a potent anti-tumor response to intracranial patient-derived xenograft tumors, justifying the clinical exploration of their efficacy in DIPG patients.

Diffuse intrinsic pontine glioma (DIPG) is an aggressive brain tumor of childhood and the leading cause of brain tumor-related death in childhood, with 2-year overall survival rates of < 10%.^1^ The recent 2021 World Health Organization’s classification of CNS tumors categorized DIPG as a subset of diffuse midline glioma (DMG), a group of tumors characterized by a diffuse growth pattern and midline location, including the spinal cord, thalamus and brainstem.^2^ Due to the location and diffuse nature of the tumor in the brainstem surgical resection is futile, with radiation treatment offering only a temporary reprieve for patients.^3^ Despite more than 60 years of clinical trials utilizing conventional chemotherapy and more recently, small molecule inhibitors, outcomes remain almost universally fatal.^4^ Given these poor outcomes, the field has shifted to utilizing more novel therapeutic approaches such as immunotherapy, to tackle this incurable disease.^5^

In part, the inability of drugs and monoclonal antibodies to sufficiently cross the blood-brain barrier has hindered treatments for brain cancer, combined with the vast heterogeneity of these poor survival cancers. The low mutational burden of DIPG also leads to an immunologically “cold” tumor microenvironment and likely contributes to the poor response to checkpoint inhibition.^5^ Some somatic mutations have been identified, including *TP53, PDGFRA, ACVR1, MYC* and the PI3K pathway, and combination targeting may provide future therapies.^6^ Promisingly, identified tumor targets expressed at the cell surface can be targeted by antigen-specific T cells which can effectively infiltrate the blood-brain barrier into the cerebrospinal fluid, even when delivered intravenously.^7^ Chimeric antigen receptors (CARs) are synthetic receptors that have the capacity to redirect endogenous T cell specificity via an antibody binding domain to recognize the cancer cell. CAR T cell therapy has been employed with great success in childhood haematological malignancies targeting CD19.^8–10^ However, the success of CAR T cell therapy in adult high-grade glioma has been disappointing, in part due to the immunosuppressive nature of gliomas, but mostly due to the heterogeneity of CNS tumors and the relative paucity of authentic tumor-specific antigens to enable sufficient tumor coverage.^5,11^

CAR T cell immunotherapy is only recently being applied for DMG patients, using tumor-targeted GD2 CAR T cells. Preclinical studies examining the efficacy of GD2 CAR T cells in DMG,^12^ led to a first-in-human phase I clinical trial (NCT04196413) in which it was demonstrated that GD2 CAR T cells could be delivered safely both intravenously and intraventricularly.^13^ Importantly, GD2 CAR T cells were effective, with 3 of the 4 reported patients showing both clinical and radiographic improvement. These early results are the first study to demonstrate the efficacy of CAR T cells in DMG,^13^ and underscore the promise and utility of CAR T cells beyond haematological cancers. However, patients eventually succumbed to their disease, and future studies are likely to require a combination of targeting approaches. Thus identifying other viable targets for CAR therapy for DIPG and other H3K27M mutated DMG is crucial to expand the applicability of this promising therapy for pediatric brain cancer patients.

With this in mind, we sought to identify additional CAR T cell targets in DIPG combining cell surface proteomics,^14^ with RNAseq and validated human epidermal growth factor receptor 2 (HER2), an oncogene, as an appropriate target for DMG/DIPG recently shown to be expressed in patients with DIPG.^15^ Amplification and overexpression of HER2 has been reported in many cancers including breast and ovarian cancers and have been well explored in glioma^16,17^ leading to it being considered a pan-cancer target for many indications. A study of tissue samples from 297 pediatric patients with nervous system tumors and rhabdomyosarcoma found that ERBB2 pathway dysregulation was identified in 20–30% of tumor types, though only 4% of the 24 DIPG samples were found to have aberrant ERBB2 pathway activation.^15^ Furthermore, Vitanza and colleagues^18^ have recently shown locoregional delivery of balanced CD4:CD8 HER2 CAR T cells for pediatric brain cancer, with demonstrable safety outcomes in three patients (with anaplastic astrocytoma and ependymoma) enrolled on the BrainChild-01 clinical trial (NCT03500991). This trial is under active clinical investigation and currently recruiting pediatric patients with medulloblastoma, ependymoma and DMG, but with localization to the pons as an exclusion criterion.^18,19^

Therefore, to explore the efficacy of HER2 CAR T cells in DIPG we generated and functionally validated both CD4^+^ and CD8^+^ CD28ζ second-generation HER2 CAR T cells. Here we demonstrate they can recognize patient-derived DIPG cells to secrete cytokines, as well as being highly cytotoxic against DIPG patient-derived cells in culture. Furthermore, the adoptive transfer of HER2 CAR T cells to treat intracranial DIPG patient-derived xenograft tumors was highly effective. In this manuscript, we demonstrate the utility and efficacy of HER2 CAR T cells in DIPG and argue that these findings warrant clinical investigation to provide additional therapeutic interventions for this devastating disease.

## Methods

### Patient-Derived Xenograft Models

Patient-derived DIPG models^20^ were a kind gift from Dr. Michelle Monje (Stanford University School of Medicine) and verified as Mycoplasma negative by PCR analysis at the WEHI internal facility. DIPG PDX cells were transduced and selected to express GFP-Firefly Luciferase for *in vivo* use (designated DIPG-GFP-Luc). DIPG PDX cells were cultured in 1:1 ratio of D-MEM/F-12 (Invitrogen) and Neurobasal^TM^-A medium (Invitrogen) supplemented with 10 mM Hepes (Gibco), 1 mM sodium pyruvate (Gibco), 0.1 mM MEM non-essential amino acids (Gibco), 2 mM GlutaMAX-I (Gibco), antibiotic-antimycotic (Gibco), B27 (Invitrogen), 20 ng/mL epidermal growth factor (EGF, Shenandoah Biotech), 20 ng/mL fibroblast growth factor (FGF, Shenandoah Biotech), 10 ng/mL platelet-derived growth factor (PDGF)-AA, (Shenandoah Biotech) and 10 ng/mL PDGF-BB (Shenandoah Biotech) and 2 μg/mL Heparin (StemCell Technologies). All cells were cultured and maintained at 37°C, 5% CO_2_.

### Cell Culture

HEK293T cells were obtained from within WEHI and cultured in RPMI-1640 (GIBCO) supplemented with 10% heat-inactivated fetal calf serum (Bovogen), 2 mMol/L GlutaMAX-I (Gibco), and penicillin-streptomycin (Gibco). All cells were cultured and maintained at 37°C, 5% CO_2_.

### Primary T Cell Culture

All human T cell experiments were conducted under WEHI HREC project 17/01LR ethical approval. Specifically, CD4^+^ negative selection (EasySep, StemCell Technologies) and CD8^+^ positive selection (EasySep, StemCell Technologies) was performed on healthy donor human PBMC isolated by Ficoll gradient separation from blood Buffy coats acquired from the Australian Red Cross Lifeblood (Agreement #21-07VIC-12). T cells were activated for 24–48 h with anti-human CD3/CD28 Dynabeads™ (Invitrogen, Catalogue #11453D) according to manufacturer’s instructions and T cells were cultured at 0.5–1 × 10^6^ cells/mL in T cell medium (RPMI-1640 (Gibco) supplemented with 10% FCS (Bovogen), 1 mmol/L sodium pyruvate (Gibco), 2 mM GlutaMAX-I (Gibco), 0.1 mM non-essential amino acids (Gibco), 50 μM Beta-mercaptoethanol (Sigma) penicillin-streptomycin (Gibco) and 50–100 IU/ml rhIL-2 (Peprotech, #200-02). Cells were maintained at 37°C, 5% CO_2_.

### Cell Surface Mass Spectrometry Analysis

Cell surface proteins were enriched and analyzed by mass spectrometry as previously described.^14^ Briefly, live cells were surface protein labeled with biotin through oxidation of terminal sialic acids on glycans with aminooxy-biotin (Biotium, USA). Cells were then lysed and streptavidin pulldown was performed using streptavidin agarose beads (Pierce, USA). On-bead digestion with trypsin (Promega) was performed and peptides were analyzed using an Impact II mass spectrometer for data acquisition and MaxQuant (ver 1.5.8.3) for data analysis.

### CAR Design and Generation of CAR T Cells

The anti-HER2 clone FRP5 scFv was obtained by accessing the publicly available scFv sequence and based on the original construct.^21^ The scFv nucleotide sequences were optimized from the protein sequence, a MYC-tag was added and the scFv was then cloned into lentivirus containing a second-generation CAR comprising of human CD8α linker, human CD28 transmembrane domain, human CD28 co-stimulation signaling domain, and a human CD3ξ ITAM containing signaling domain. Lentivirus was produced in HEK293T cells transfected with pMD2G-VSVg (Addgene #12259), pMDLg/pRRE (Addgene #12251), pRSV-REV (Addgene #12253) and the CAR lentiviral transfer vector, using FuGENE-6 (Promega, Catalogue Number E269A). Viral supernatant was used to transduce activated T cells.

### Flow Cytometry

Samples were incubated with antibodies diluted in PBS containing 0.2% FCS (FACS buffer) for 30 min at 4°C before samples were analyzed using a Fortessa X20 (BD Biosciences) and analysis was performed using FlowJo software (Becton Dickinson). Gating strategy is shown in Supplementary Figure 1. Antibodies used include anti-MYCTag-AF488 (Clone 9B11, Cell Signaling Technology), anti-CD25-APC (Clone PC61, BioLegend), anti-CD69-BV650 (Clone H1.2F3, BioLegend), anti-CD4-FITC (Clone GK1.5, produced at WEHI Bundoora), and anti-CD8-PE antibodies (Clone 53-6.7, BD Pharmingen).

### Incucyte Cytotoxicity Assay

Adherent target cells were seeded in triplicate in 96 flat bottom plates before co-incubation with effector CAR T cells at a range of effector:target ratios, and normalized for CAR expression. Cells were co-cultured in media containing 200 µM Propidium Iodide (PI, Calbiochem), and plates imaged using an IncyCyte every hour (models). Percentage lysis was determined as ((sample PI count—spontaneous PI counts)/(maximum PI count at 0-h scan) × 100).

### Cytokine Bead Array (CBA)

The cytokines IFNγ and TNFα as well as cytotoxic granule cargo granzyme B were measured from culture supernatant using a cytokine bead array (CBA), according to the manufacturer’s specifications (BD Biosciences). The CBA was assayed using flex-sets for human IFNγ, TNFα and Granzyme B. Samples were analyzed using a FACS VERSE (BD Biosciences), and analysis used FCAP array software version 3.0 (BD Biosciences).

### CAR T Cell Activation Co-culture

CAR T cell activation was assessed by flow cytometric analysis of activation markers following *in vitro* stimulation. CAR T cells and tumor cells were co-incubated for 10 h in T cell media at a 1:1 ratio at 37°C and 5% CO_2_, in triplicate. Positive control for CAR stimulation was provided using plate-bound unconjugated anti-MYC-tag (Clone 9B11, Cell Signaling Technology), pre-coated overnight at 4°C at 1:1000 in PBS. After co-culture, the cells were assayed for the expression of activation markers.

### Mice

All mice used were female 6–8 weeks old NOD.Cg-Prkdc^scid^IL2rg^tmWjl^/SzJ Charles River Altered Schnaedler Flora mice (NSG), sourced from the WEHI animal facility (Kew, Victoria, Australia). They were bred and maintained under specific pathogen-free conditions in these facilities and transferred to WEHI animal facilities (Parkville, Victoria). All experiments were performed with the approval of the WEHI Animal Ethics Committee (approval 2019.020). To establish intracranial tumors, 5 × 10^4^ SU-DIPG36-GFP-Luc cells were implanted intracranially at 2 mm right of bregma at the coronal suture to a depth of 3 mm from the cranium into the brain, using a stereotactic frame. Mice were monitored according to ethics approval 2019.020. To assign mice to treatment groups they were ranked by tumor size and assigned evenly with a negative bias to CAR treatment. CAR-treated mice were injected intravenously via the tail vein with 1:1 CD4^+^:CD8^+^ T cells.

### In Vivo Imaging

Intracranial SU-DIPG36-GFP-Luc tumor-bearing mice were assessed weekly by injecting IP with 3 mg VivoGlo Luciferin (Promega, P1043) before bioluminescence imaging (BLI) to determine with tumor growth IVIS Lumina III Series Hardware (Perkin Elmer). Images were captured and quantified as average radiance (p/s/cm^2^/sr). Data were analyzed using Living Image software (Perkin Elmer).

### Histology

Brains from xenograft models were fixed in 10% formalin and embedded in paraffin. Sections were cut and stained with Hematoxylin and Eosin (H&E), anti-CD4 (ab133616, Abcam) anti-CD8 (MA5-14548, Invitrogen) or anti-GFP (ab6556, Abcam) antibodies. Sections were then scanned with a PANORAMIC scan II (3DHISTECH) and images were analyzed with QuPath.^22^

### Statistics

Statistical analyses were performed using GraphPad Prism 8 software. Statistical tests applied include Mann–Whitney and two-way ANOVA with *post hoc* analysis using Tukey’s multiple comparisons. Asterisks within figures refer to statistical differences between test and control groups, *P* values and the number of replicate experiments performed to derive the data are indicated in the figure legends.

## Results

### HER2 is Highly Expressed by DIPG Tumors

**Figure 1.**
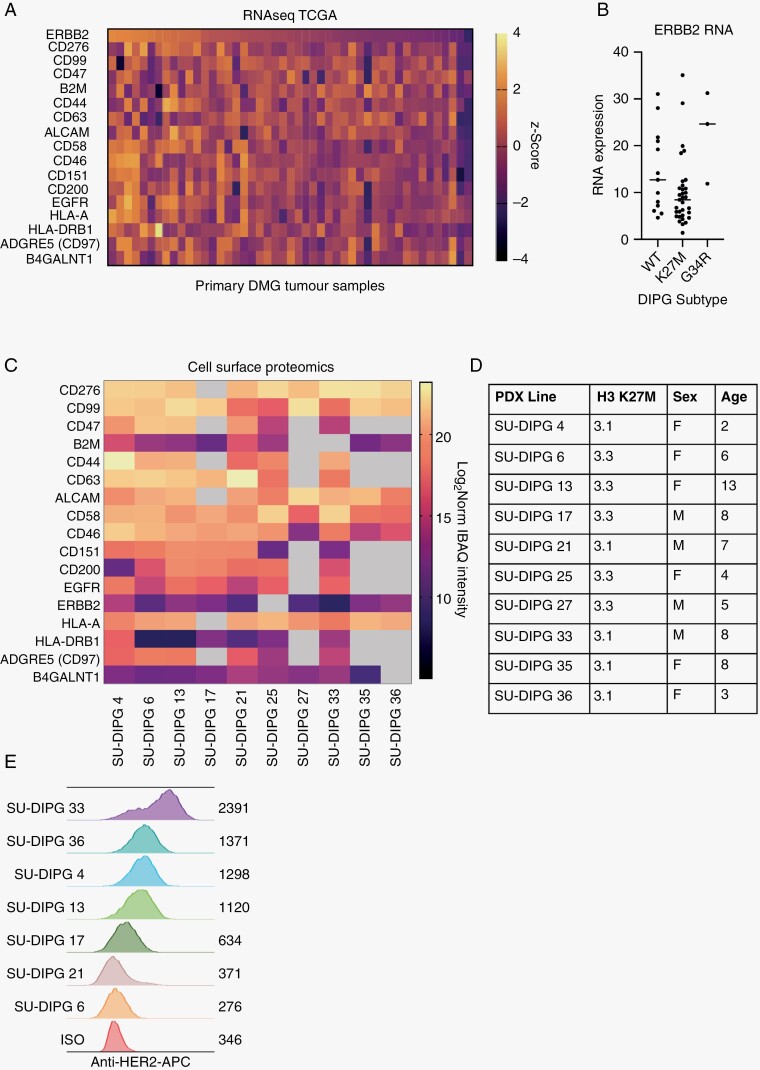
HER2 protein is highly expressed by DMG primary tumors and DIPG patient-derived models. (A) Primary DMG tumor RNA seq (*z*-score) from the TCGA dataset indicating high to low mRNA expression, ranked by HER2 expression between patient samples. (B) RNA expression for ERBB2 across WT, K27M or G34R DIPG patients from the TCGA dataset. (C) Cell surface proteomics of 10 patient-derived DIPG models shows a selection of relevant proteins detected at the cell surface, grey indicates no detection (*n* = 4 technical replicates/model). (D) Table indicating DIPG PDX model particulars including H3K27M status, sex and age. (E) Representative flow cytometry histograms of DIPG cells labeled with anti-HER2-APC antibody ranked in descending order of gMFI (listed on right) compared to isotype control staining of SU-DIPG36.

RNA-sequencing data from 47 patients (pts) within the TCGA dataset was analyzed for the expression of known targets previously identified in DIPG (Figure 1A).^12,23,24^ From this dataset, we determined that 27.6% (13 pts) express high HER2 transcript (*ERBB2)* and 63.8% (30 pts) had detectable RNA in their tumors. Ranking the dataset based on patients with high HER2 expression also identified multiple targets that may be co-expressed on a subset of DIPG patients. Here we identified the co-expression of the known CAR targets EGFR and CD276 as well as the macrophage “don’t eat me” signal CD47. Unlike some antigens including GD2 which are restricted to H3K27M-altered tumors, HER2 gene expression is highly expressed across DMG/DIPG H3 mutant, as well as K27M WT tumors indicating that targeting HER2 in DIPG will be broadly applicable (Figure 1B). In addition, HER2 gene expression is noted in G34R mutated hemispheric gliomas, indicating that HER2 could also be targeted in this disease entity.

To determine the surface expression of proteins from potential targets identified in the RNAseq TCGA dataset, we next performed cell surface mass spectrometry on 10 DIPG patient-derived xenograft (PDX). The data was analyzed for the presence of known tumor-associated antigens previously detected on DIPG PDX models (Figure 1C).^12^ All 10 DIPG PDX Models were H3 K27M mutated and covered both male and female patients (Figure 1D). Our in-depth analysis of the cell surface proteome of DIPG PDX models shows diversity in abundance between the detected proteins as measured by IBAQ (a score of intensity divided by protein size). As has been previously reported CD276 (also known as B7-H3) is an abundant protein highly expressed on the surface of all ten DIPG PDX models tested. Previous studies by Mackall and colleagues^25^ have shown CD276 to also be an ideal CAR target with preclinical activity demonstrated against pediatric medulloblastoma and DMG tumors. This is the focus of BrainChild-03 clinical trial currently recruiting from Seattle Children’s Hospital (NCT04185038).

Relatively high HER2 abundance measured by cell surface proteomics holds significant translational potential in DIPG as a safe validated target.^18^ Therefore, we sought to determine how IBAQ HER2 detection by cell surface mass spectrometry compared to flow cytometry. To achieve this, we measured HER2 expression on a panel of 7 SU-DIPG PDX models by flow cytometry (Figure 1E and Supplementary Figure 1). Interestingly, we were able to detect HER2 expression by flow cytometry in all but one of the DIPG PDX models interrogated. These data confirm that HER2 expression is broadly expressed by DIPG PDX models, with the highest expression detected on SU-DIPG33 and SU-DIPG36 with mean fluorescent intensities (MFI) more than 3- and 6-fold higher, respectively, than HER2-negative DIPG6 cells (Figure 1E). SU-DIPG13 and SU-DIPG17, which are H3.3K27M mutated, also have similar HER2 expression to SU-DIPG36, which is H3.1K27M mutated, therefore HER2 expression is independent of the genetic subtype. Within the TCGA dataset, 63% (31/49) of patients showed mRNA expression for HER2 with 71% (5/7) of the PDX DIPG cell lines also expressing HER2 (Figure 1A and E).

### HER2 CAR T Cells are Activated by SU-DIPG33 and SU-DIPG36 PDX Cells

**Figure 2.**
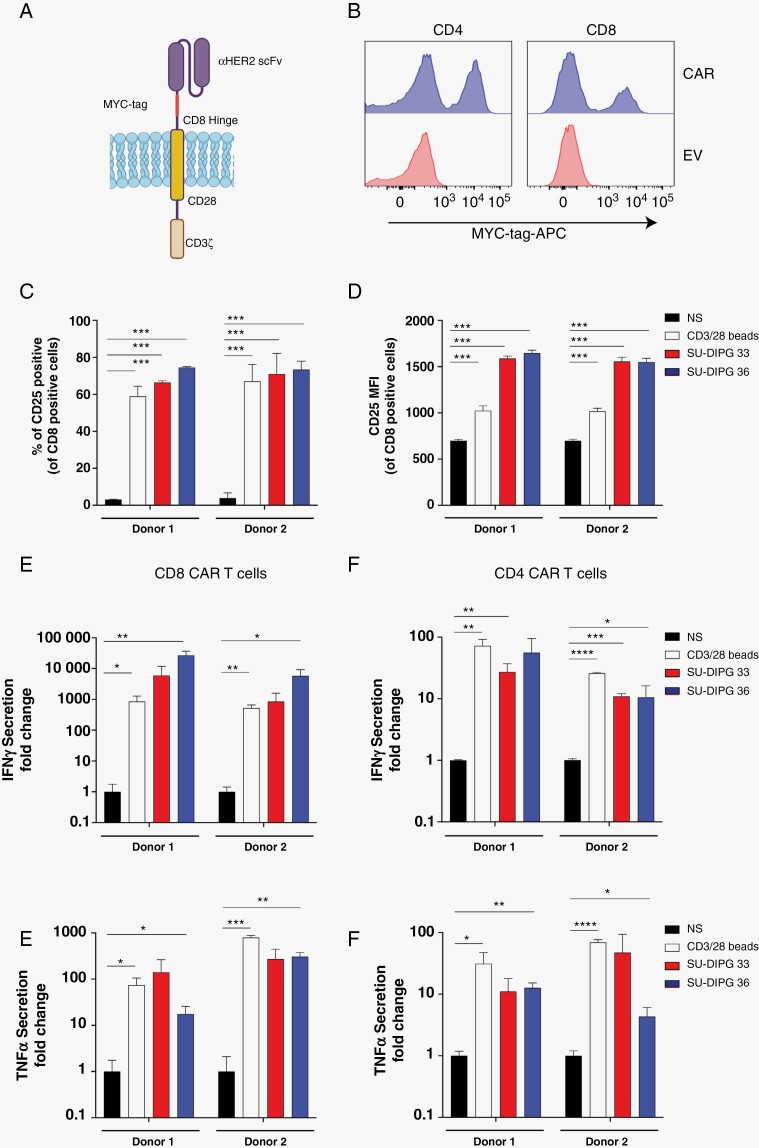
Both CD4^+^ and CD8^+^ HER2 CAR T cells recognize both SU-DIPG33 and SU-DIPG36 cells in an antigen-dependent manner to upregulate activation markers and secrete cytokine. (A) Schematic of second-generation CD28ζ HER2 CAR (clone FRP5) used in the study under an EF1α promoter. Representative CAR cell surface detection via anti-MYC-tag-APC in both human CD4^+^ and CD8^+^ T cells (B) compared to empty vector transduced controls. Detection of activation marker CD25 as a percentage of positive cells (C) or gMFI (D) when CD8^+^ HER2 CAR T cells were co-cultured with either SU-DIPG33 or SU-DIPG36 at 1:1, or CD3ε/CD28 beads at 1:1 from two different PBMC donors. Both CD8^+^ and CD4^+^ HER2 CAR T cells were co-incubated with either SU-DIPG33 or SU-DIPG36 at 1:1, or CD3ε/CD28 beads at 1:1 and supernatants probed for IFNγ (E, F) or TNFα (G, H) levels by CBA and normalized to the mean fold-change in cytokine production over non-stimulated cells (mean ± *SD*). Statistical significance between groups was calculated by comparing individual groups using students’ nonparametric *T*-test. ^*^P < .05, ^**^P < .001, ^***^P < .0001, ^****^P < .00001.

Having validated HER2 as a potential therapeutic target in DIPG, we then looked to determine the efficacy of targeting DIPG cells with HER2 CAR T cells. To do this a second-generation CAR vector was employed using the HER2-specific scFv clone FRP5 with a CD28-CD3ζ signaling tail (Figure 2A).^21^ To determine the cell surface expression of the HER2 CAR, a MYC-tag was inserted into the linker domain (Figure 2A). We then purified human CD4^+^ and CD8^+^ T cells, transduced them with the HER2 CAR and measured cell surface expression via MYC-tag binding (Figure 2B). Clear CAR-positive populations were detected in both CD8^+^ and CD4^+^ T cells via surface-bound anti-MYC-tag antibody labeling (Figure 2B).

Expression of the IL-2Rα chain CD25 is a hallmark of antigen-specific T cell activation. To assess the capacity of human CD8^+^ HER2 CAR T cells to be activated by HER2 expressing DIPG cells, two independent PMBC donors were co-cultured at 37°C with SU-DIPG33 or SU-DIPG36 cells for 5 h. The HER2 CAR T cells generated from both human donors demonstrated significant upregulation of CD25 with an increase in percentage positive cells and MFI when co-cultured with either DIPG PDX model (Figure 2C and D). The observed increased CD25 was similar to the percentage increase of CD25 detected in the *α*CD3/CD28 bead positive control, indicating that the CAR induced a strong response. In addition, there were statistically significant increases in CD25 MFI when the HER2 CAR T cells were co-cultured with DIPG cells above the *α*CD3/CD28 bead positive control (Figure 2D). Taken together, these data show a robust HER2 CAR T cell activation to DIPG cells.

### CD4^+^ and CD8^+^ HER2 CAR T Cells Functionally Recognize SU-DIPG33 and SU-DIPG36 PDX Cells

Having established that HER2 CAR T cells recognize and are activated by DIPG PDX models, we then wanted to determine the function of the HER2 CAR in CD4^+^ and CD8^+^ T cells. To do this we functionally assessed for IFN*γ* and TNF*α* secretion by both CD4 and CD8 HER2 CAR T cells in a co-culture assay with SU-DIPG33 and SU-DIPG36 (Figure 2E–H). We found significant increases in IFN*γ* when either CD4^+^ or CD8^+^ HER2 CAR T cells were co-cultured with both SU-DIPG33 and SU-DIPG36, compared to non-stimulated cells (Figure 2E and F). Compared to aCD3/CD28 bead positive control we also observed a significant increase in IFN*γ* in CD8^+^ but not CD4^+^ HER2 CAR T cells (Figure 2E). These data are reflective of the orders of magnitude higher (10–100 fold) IFN*γ* secreted by CD8^+^ HER2 CAR T cells as the primary source of IFN*γ* in an immune response. Similarly, TNF*α* secretion was also secreted by HER2 CAR T cells specifically in response to DIPG PDX cells, (Figure 2G and H). These data demonstrate that both CD4^+^ and CD8^+^ HER2 CAR T recognize and are capable of robust effector molecule secretion in response to DIPG PDX cells.

### CD8^+^ HER2 CAR T Cells are Cytotoxic to SU-DIPG36 PDX Cells

**Figure 3.**
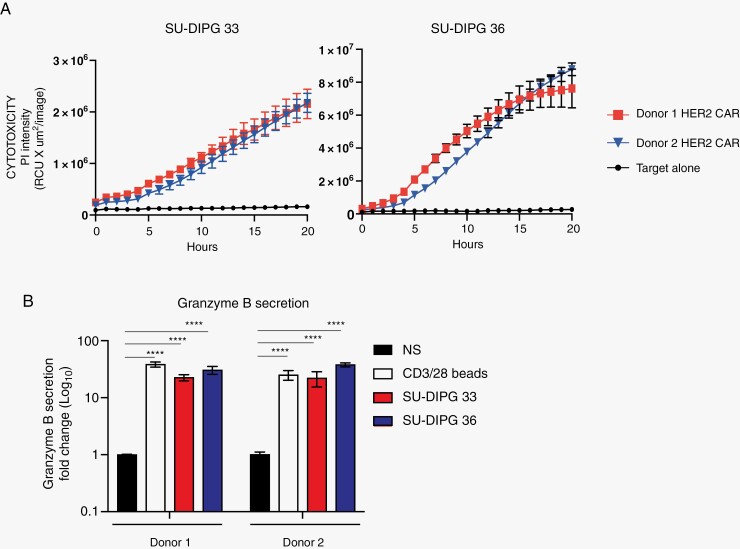
HER2 CAR T cells kill SU-DIPG36 tumor cells *in vitro*. CD8^+^ T cells from 2 separate human donors were transduced with HER2 CARs and co-cultured with SU-DIPG33 or SU-DIPG36 cells for 20 h. (A) Target cell death as determined by uptake of PI over time. Black circles represent spontaneous death from targets alone. (B) Secretion of granzyme B after 20 h of stimulation by *α*CD3/28 beads, SU-DIPG33 and SU-DIPG36 cells, as detected by cytokine bead array from the culture supernatant. Shown is the fold-change over non-stimulated (NS) across 2 human donors (error bars show *SD* from triplicate wells). ^****^P > .0001 Two-way ANOVA with TUKEY’S.

Having established that CD8^+^ HER2 CAR T recognize DIPG PDX cells, we next examined cytotoxicity. We examined the capacity of CD8^+^ HER2 CAR T cell killing of SU-DIPG36 cells using an Incucyte killing assay. CD8^+^ HER2 CAR T cells from two donors were co-cultured with SU-DIPG36 in media containing propidium iodide (PI) and imaged every hour in an Incucyte imaging platform to provide kinetic cytotoxicity monitoring. The uptake of PI over time into target cells was used as a surrogate of DIPG cell death. As expected, CD8^+^ HER2 CAR T cells from both donors demonstrated potent and efficient target cell death, with minimal cell death observed in target-alone cultures (Figure 3A). Given the capacity for CD8^+^ HER2 CAR T cells to kill SU-DIPG36, we next examined the release of granzyme B when co-cultured with SU-DIPG33 or SU-DIPG36 cells (Figure 3B). Both SU-DIPG33 and SU-DIPG36 induced degranulation and granzyme B secretion > 10-fold over baseline, similar to *α*CD3/CD28 bead positive controls, when co-cultured with CD8 + HER2 CAR T cells (Figure 3B). These data conclusively show that CD8^+^ HER2 CAR T cells are highly capable of killing DIPG cells *in vitro*.

### HER2 CAR T Cells are a Robust Therapy Against Intracranial SU-DIPG36 PDX Tumors

**Figure 4.**
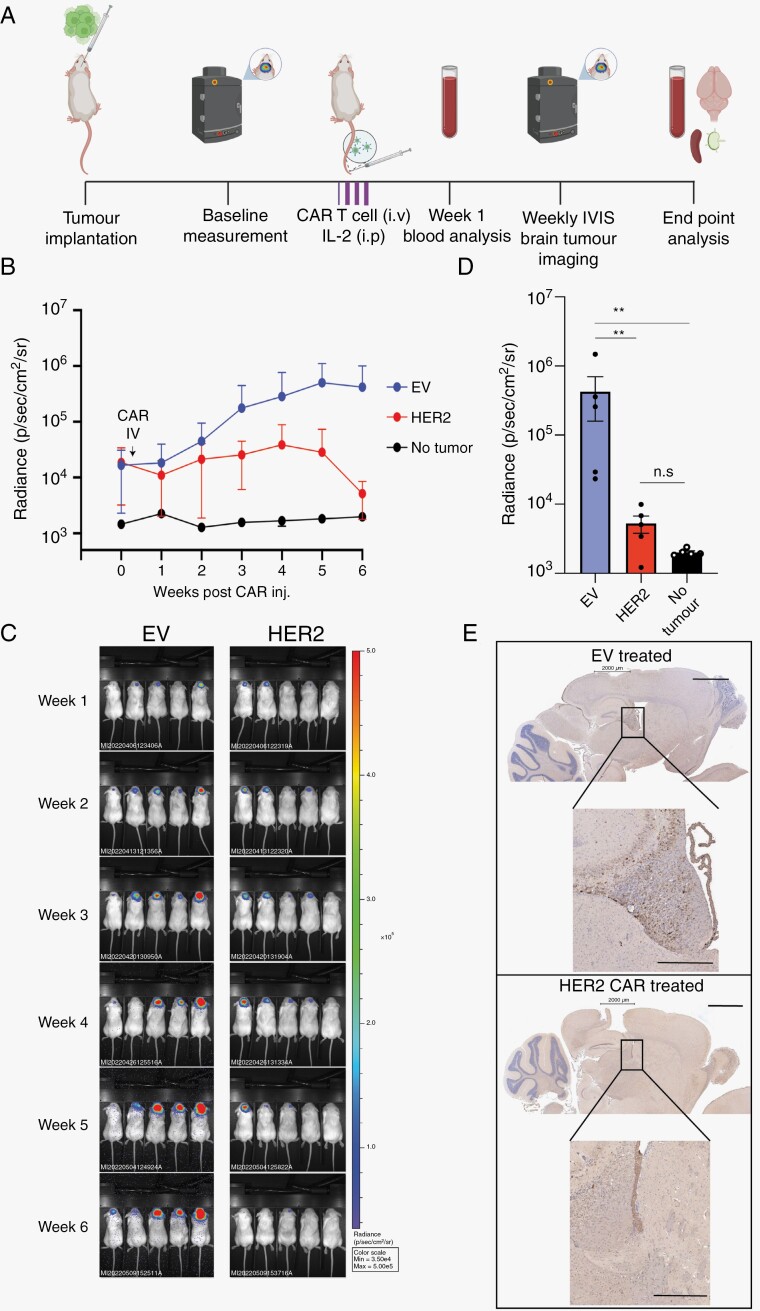
HER2 CAR T cells adoptively transferred into SU-DIPG36 intracranial tumor-bearing NSG mice. (A) Schematic to show the HER2 intracranial CAR T cell tumor model. SU-DIPG36-GFP-Luc cells were injected intracranially into NSG mice and stratified into treatment group according to tumor size 1 week after injection. Two weeks after tumor implantation, No Tumour (NT) or 10^6^ human CAR T or EV cells at a 1:1 CD4+:CD8+ were intravenously injected through the tail vein. Mice were injected *i.p* with 50e4IU of IL-2 on the day of CAR therapy and for 3 days following T cell infusion and IVIS was imaged weekly for tumor size. (B) Graphed averages of tumor size as determined by IVIS imaging and luciferase activity. Shown is the mean radiance (p/s/cm^2^/sr) of mice 6 weeks (± *SD*) after CAR T injection (C) Images of the mice with luciferase detection superimposed. (D) BLI measurements at the endpoint for each group as shown in (B). Error bars depict ± *SD n* = 5 mice per group, n.s = not significant, ^**^0.079 Mann–Whitney Test. (E) Endpoint (6 weeks post CAR T cell infusion) images of representative of SU-DIPG 36 tumor-bearing mouse brains showing GFP+ tumor cells (brown). Panels on the left show representative control mouse (injected with SU-DIPG36-GFP-Luc tumor cells and treated with an EV control CAR T cells), panels on the right show mouse injected with DIPG36-GFP-Luc tumor cells and treated with HER2 specific CAR T cells. Scale bar is 2 mm for the top panels and 0.5 mm for the bottom panels.

Having demonstrated that HER2 CAR T cells are highly effective at producing cytokines and killing DIPG PDX cells when co-cultured *in vitro* we then sought to explore their function *in vivo*. To do this we established intracranial SU-DIPG36-GFP-Luciferase^+^ tumors in NSG mice (Figure 4A). Following implantation, tumor size was determined using bioluminescent IVIS imaging and mice were then assigned to treatment groups of either 1:1 CD4^+^:CD8^+^ human HER2 CAR T cells or negative control Empty Vector (EV) human T cells (Supplementary Figure 1). Following intravenous T cell infusion, mice were given daily IL-2 IP injections for three days to support engraftment. One-week post-transfer mice were bled, and flow cytometry was used to detect circulating CAR T cells and determine T cell engraftment. All mice showed both CD4^+^ and CD8^+^ T cells in circulation (Supplementary Figure 2). Interestingly, a significantly greater number of human CAR T cells were discovered in mice that received the HER2-CAR, rather than EV controls.

To determine the effect of HER2 CAR T cell treatment on tumor growth, intracranial tumor-bearing mice were measured weekly using bioluminescent IVIS imaging (Figure 4B and C). HER2 CAR T cell treatment led to a significant reduction in tumor signal compared to EV T cells, with HER2 CAR T cell-treated mice returning to baseline six weeks post-transfer (Figure 4B). Images showing BLI overlays on mice imaged weekly showed a reduction in tumors detected in the brain at Week 6 post CAR infusion (Figure 4C). Endpoint analysis of tumor expression at Week 6 post CAR infusion showed a statistically significant decrease in tumors detected in HER2 CAR-treated mice when compared to EV controls. Importantly there was no significant difference in the detection of tumors between HER2 CAR-treated mice and mice that did not receive tumors, indicating either that tumor had been eradicated or that it was below the range of detection. Histological analysis of mouse brains at endpoint showed tumor burden in the EV treated mice compared either no tumor burden or low level residual tumor cells in the HER2 CAR T cell-treated mice. Therefore, HER2 CAR T cell treatment robustly decreased tumor burden and at endpoint, tumors were still regressing and but small numbers of tumor cells were observed at this timepoint (Figure 4E).

**Figure 5.**
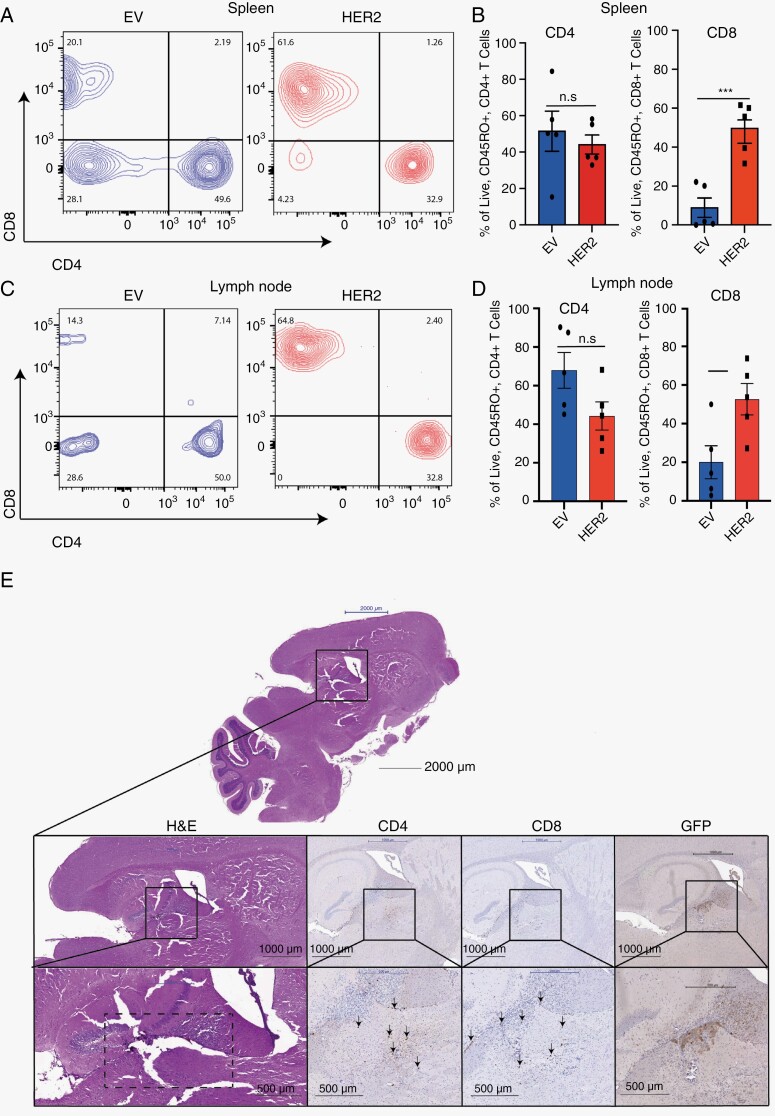
HER2 CAR T cells persist in the brain, spleen and lymph nodes of recipient mice. Representative flow cytometry of live CD45RO^+^ cells from spleen (A) and lymph node (C) showing CD4^+^ and CD8^+^ CAR T cell engraftment in NSG SU-DIPG36 PDX tumor-bearing mice (*n* = 5 per group) week 6 post CAR infusion (experimental endpoint). The mean percentage of live CD4^+^ and CD8^+^ CART cells is quantified from spleen (B) and lymph nodes (D) and shown is the mean % cells ± SEM for the individual 5 mice. n.s = not significant, ^*^P = .025, ^***^P = .001 non parametric Student’s *t*-test. (E) Endpoint (6 weeks post CAR T cell infusion) H&E staining of representative mouse brains from a SU-DIPG36 tumor-bearing mouse treated with HER2-specific CAR T cells show infiltration of human CD4^+^ and CD8^+^ cells (black arrows) and some small numbers of GFP + tumor cells. Staining was performed with anti-CD4 (ab133616, Abcam, 1/500), anti-CD8 (MA5-14548, Invitrogen, 1/100) or anti-GFP (ab6556), Histofine Simple Stain MAX (Nichirei Biosciences Inc.) secondary antibodies conjugated to peroxidase were then used and signal was developed with diaminobenzidine followed by counterstaining with hematoxylin. Scale bar is 1 mm in top image and 0.5 mm in bottom panel.

Analysis of the spleen and blood by flow cytometry showed an expansion of CD8^+^ T cells that only occurred in the HER2-CAR T cell-treated mice and not EV CAR T cell-treated controls, suggesting an antigen-specific CAR T cell expansion may have occurred (Figure 5A, B and Supplementary Figure 2). CAR T cell persistence was also observed in the lymph nodes of the treated mice (Figure 5C and D). As NSG mice lack CD4 and CD8 T cells we investigated CAR T cell infiltration into the mouse brains via H&E histology of the whole brain from CAR T cell or EV T cell-treated mice and probed for human CD4^+^ or CD8^+^ expression using clinically relevant and specific anti-human antibodies. We observed a robust population of CD4^+^ and CD8^+^ T cells detected in the brains of HER2 CAR treated animals (Figure 5E) but not EV control mice (Supplementary Figure 3). Together these data show that HER2 CAR T cells provide a robust anti-tumor response in SU-DIPG36 facilitated by strong CD4^+^ and CD8^+^ T cell engraftment and infiltration of the brain. Histology H&E staining suggested a large presence of tumor cells in the EV controls (Supplementary Figure 3) as denoted by nuclear density but a vastly reduced tumor burden in HER2 CAR treated mice (Figure 5E). It cannot be ruled out, however, that undetectable numbers of tumor cells may still be present in HER2 CAR T cell-treated brains and in one mouse we detected some residual HER2 + tumor cells at 6 weeks post CART cell administration (Figure 5E) and due to the termination of experiments after 7 weeks due to graft versus host disease, long-term outgrowth of tumor cells cannot be ruled out.

## Discussion

Here, we have described the efficacy of HER2 CAR T cells *in vitro* and *in vivo* against patient-derived DIPG models that all contain the H3K27M mutation. However, we also show that within the TCGA dataset high HER2 is not restricted to histone mutant DIPG. These data demonstrate that the HER2 CAR can recognize endogenous levels of HER2 expressed on patient-derived DIPG models resulting in both cytotoxicity and cytokine production. Furthermore, like GD2 CAR T cells,^12^ HER2 CAR T cells are capable of controlling intracranial patient-derived DIPG xenograft tumors with T cells identified in the brain parenchyma, illustrating their successful crossing of the blood-brain barrier. HER2 CARs demonstrated persistence in the blood, spleen and lymph nodes of treated mice, notable for CARs that utilize a CD28 co-stimulation domain which has been implicated in decreased persistence clinically.^26^ Whilst we observed persistence of CD4^+^ T cells in both EV and CAR T cell-treated mice in the lymph node and spleen, only CD8^+^ T cells specific to HER2 persisted suggesting a stronger antigen-dependent mechanism for persistence for CD8^+^ T cells.

Our data demonstrate an impressive reduction in tumor burden below the detection range of BLI imaging; however, we cannot rule out the possibility of residual tumor burden. We know from collective experience with CAR T therapy that targeting a single antigen, both preclinically and clinically, is unlikely to yield a durable long-term remission. Even in CD19-CAR T cell targeting of Acute Lymphoblastic Leukaemia, 40% of patients relapse of which 30% were antigen-negative tumors,^27^ and therefore it is certain that combination approaches will require multitargeting of tumor antigens for heterogeneous tumors like DIPG. Within this framework, we hope that our findings can help to enhance and expand the applicability of CAR T cell therapy to benefit H3.3K27M GD2 positive patients, as well as those that are GD2 negative. In addition, our data suggests that HER2 CAR T cells can be applied to a broader range of patients than GD2 CAR T cells as HER2 expression was not restricted to K27M patients. HER2 CAR effector cells have been applied and shown to be effective in other glioma models such as glioblastoma.^16,28^

Our study is timely given a recent interim report of BrainChild-01 outlining the treatment of 3 young adults with high-grade brain tumors with HER2-specific CAR T cells administered locoregionally via CNS catheter (NCT03500991).^18^ This interim analysis demonstrates the clinical feasibility of a HER2 CAR T cell trial in a pediatric setting, with patients experiencing no dose-limiting toxicity, supporting that HER2 CAR T cells can be safely administered in the pediatric context which facilitates rapid clinical translation in DMG.^18^ It should be noted that both pediatric HER2 CAR T cell trials currently open for enrollment specifically exclude DIPG patients (NCT02442297, NCT03500991), and given our data we would suggest that the option to open these clinical trials to include appropriate HER2 high DIPG patients should be considered. The evidence presented here that targeting HER2 via CAR T cell therapy in DIPG is effective is critically important for the initiation of clinical trials to impact this extremely low survival brain cancer. In additional trials, BrainChild-02 (NCT03638167) delivers EGFR-specific CAR T cells to pediatric CNS tumor patients, and BrainChild-03 (NCT04185038), delivered CD276-specifc CAR T cells to pediatric patients with CNS tumors, including DIPG. Combined, these studies suggest that as monotherapies are shown to be safe, they may be applied in a combination strategy and additional targeting can offer a reprieve to antigen escape.

It is becoming increasingly clear that an effective CAR T therapy for brain cancer can be developed and that it is likely to need to target multiple antigens as well as be combined with other immunotherapies, such as checkpoint blockade. While checkpoint blockade using anti-CTLA-4 ipilimumab, and anti-PD1 antibodies nivolumab or pembrolizumab for DMG has failed as a monotherapy,^29^ enhancement of CAR T cell immunotherapy in combination with checkpoint blockade may improve anti-tumor efficacy. We show that in a subset of patients with high expression of HER2 there is also concordant high expression of CD276 and CD47 (Figure 1A). Both CD276 and CD47 have previously been identified as immunotherapy targets and are under active investigation, as a CAR and antibody respectively, in DMG. A CD276 CAR T cell clinical trial has opened specifically targeting a range of pediatric brain tumors including DIPG (NCT04185038) and early safety studies have shown good clinical tolerance even after 40 infusions *icv*.^30^ With the macrophage “don’t eat me signal” CD47 being highly expressed in DMG/DIPG tumors, this suggests that CAR T therapy in combination with blocking anti-CD47 antibodies may provide a potentially synergistic treatment. Indeed, CD47 monotherapy has shown preclinical promise in pediatric neuroblastoma models, synergising with GD2-CAR T cell approaches.^31^ Again, despite preclinical evidence demonstrating efficacy, DMG in the pons is an exclusion criterion in the exploration of anti-CD47 monotherapy in pediatric and adult CNS tumors (NCT05169944). Combining the HER2 CAR with GD2 CAR, CD276 CAR in combination with anti-CD47 antibody therapy may prove to be sufficiently potent to eradicate DIPG tumor cells in the brain and warrants further investigation. Previously work has shown that a tandem CAR approach targeting HER2 and IL13Rα2^32^ or trivalent CARs with Ephα2^33^ in adult glioblastoma, can help mitigate tumor antigen escape which led to a dose escalation clinical trial.^34^ Given the results from this and previous studies HER2 targeting in a multi antigen approach should be further explored in the context of more diffuse gliomas such as pediatric DIPG.

HER2 CAR T cells are part of a small but growing arsenal of potential immunotherapeutic treatments for DMG/DIPG and future work will require a focus on the combinatorial effects of targeting multiple antigens to unlock the immune system to fight DMG/DIPG. Collectively, there is an optimistic outlook for pediatric brain cancer patients and their families, with the increased identification and preclinical validation of CAR targets and other immunotherapies there is momentum building to shift clinical practice forwards and alter the outcomes for patients.

## Supplementary Material

vdad024_suppl_Supplementary_Figure_1

vdad024_suppl_Supplementary_Figure_2

vdad024_suppl_Supplementary_Figure_3

vdad024_suppl_Supplementary_Material
