# Supplementary figures and images for: HER2 chimeric antigen receptor T cell immunotherapy is an effective treatment for diffuse intrinsic pontine glioma
Source: Neurooncol Adv. 2023 May 4;5(1):vdad024. doi: 10.1093/noajnl/vdad024 (PMC10158089; doi:10.1093/noajnl/vdad024)

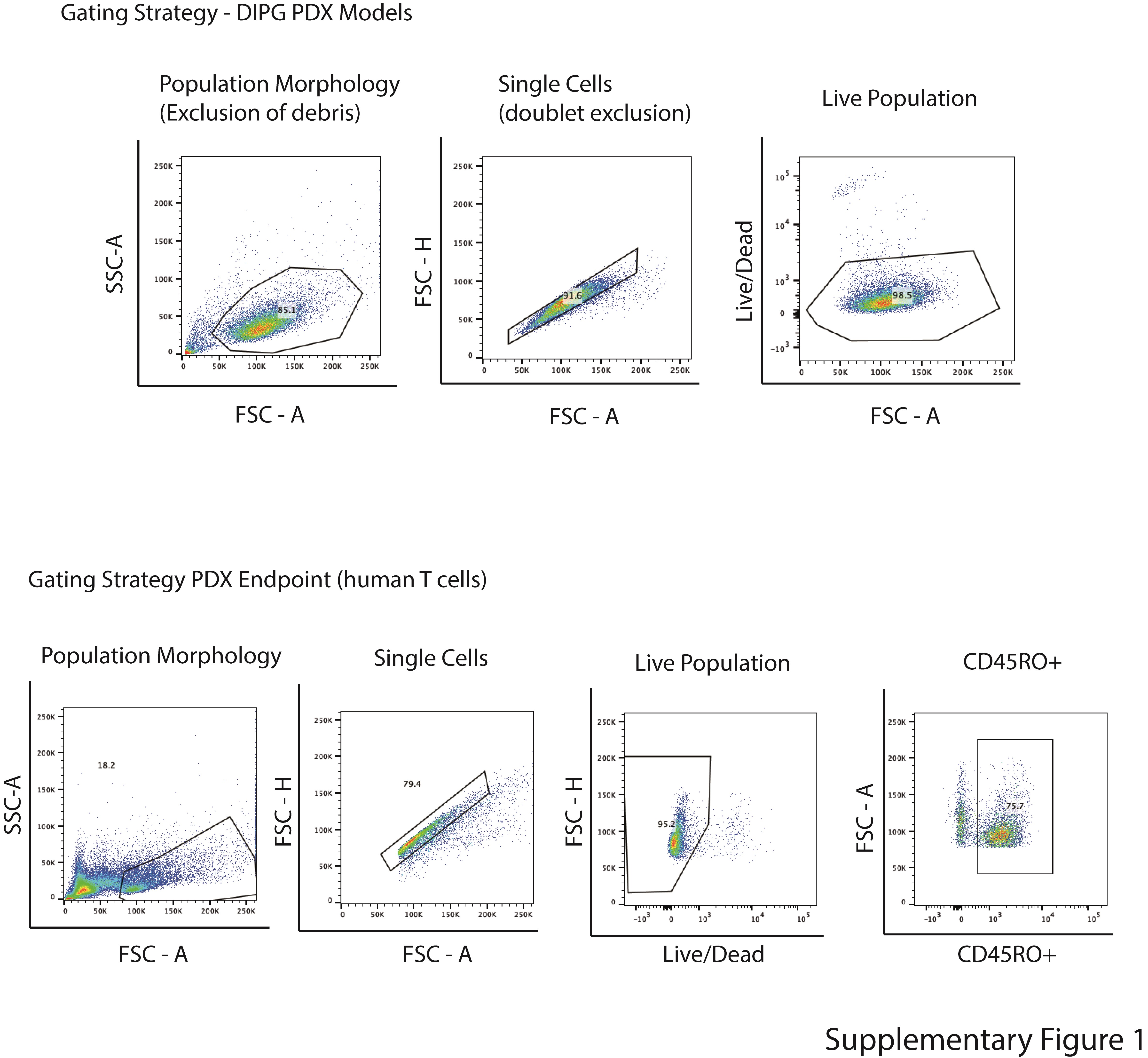

Supplement: vdad024_suppl_Supplementary_Figure_1 [file vdad024_suppl_supplementary_figure_1.jpeg]

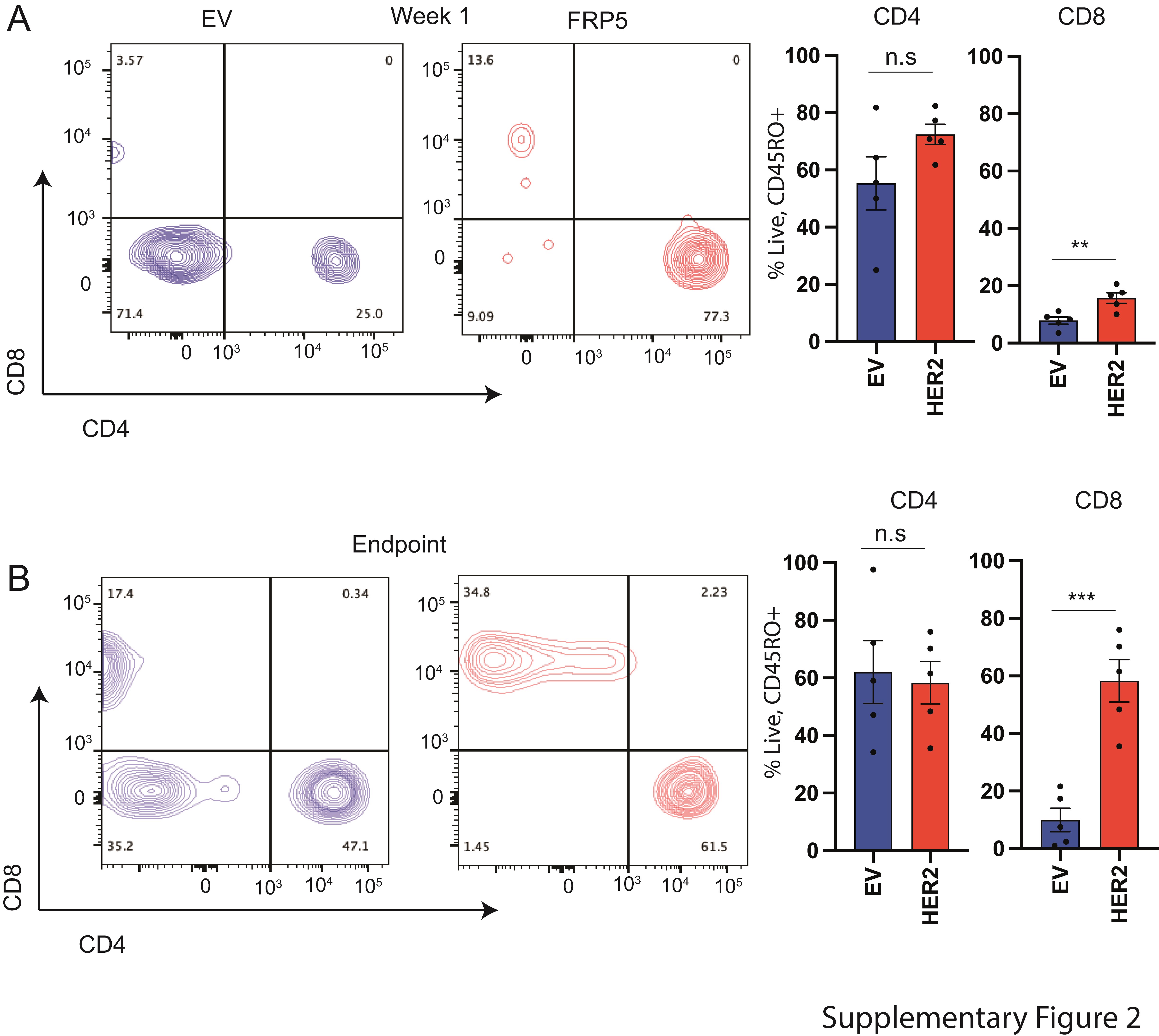

Supplement: vdad024_suppl_Supplementary_Figure_2 [file vdad024_suppl_supplementary_figure_2.jpeg]

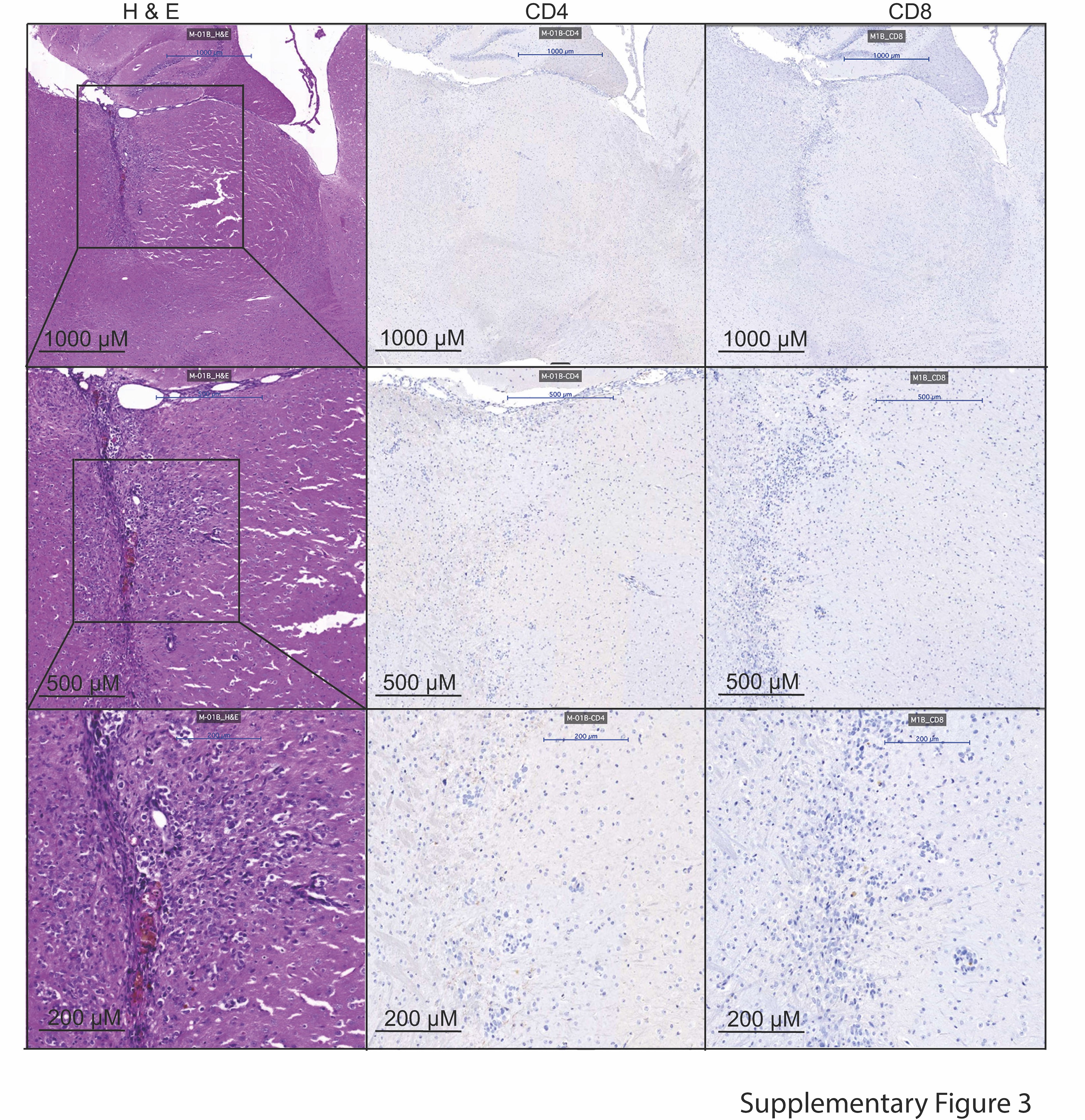

Supplement: vdad024_suppl_Supplementary_Figure_3 [file vdad024_suppl_supplementary_figure_3.jpeg]
