# Supplementary material for: HER2 chimeric antigen receptor T cell immunotherapy is an effective treatment for diffuse intrinsic pontine glioma
Source: Neurooncol Adv. 2023 May 4;5(1):vdad024. doi: 10.1093/noajnl/vdad024 (PMC10158089; doi:10.1093/noajnl/vdad024)
Supplement: vdad024_suppl_Supplementary_Material [file vdad024_suppl_supplementary_material.docx]

**Supplementary Figure Legends**

**Supplementary Figure 1:** Gating strategy for detection of HER2 on DIPG PDX cells or the detection of donor T cells from animal models.

In all instances of flow cytometry, Morphology was first detected using forward and Side Scatter gating. Doublets were excluded from analysis (based on FSC-A vs FSC-H) as were dead cells (In the case of human T cell detection from PDX mouse models, CD45RO^+^ cells were selected for further analysis.

**Supplementary Figure 2:** HER2 CAR T cells persist in the blood of DIPG36 tumour bearing mice. Representative flow cytometry of live CD45RO^+^ cells from blood week 1 post T cell transfer (A) and week 6 post CAR infusion (endpoint) (B) showing CD4^+^ and CD8^+^ CAR T cell engraftment in NSG DIPG36 PDX tumour-bearing mice (n=5 per group). The mean percentage of live CD4^+^ and CD8^+^ CART cells is quantified and shown is the mean % cells ±SEM for the individual 5 mice and gated on live, CD45RO^+^ cells. n.s = not significant,** P = 0.0073 *** P = 0.0004 non parametric Student’s t-Test.

**Supplementary Figure 3:** T cells were not detected in the brain of EV T cell treated mice.

H&E staining of EV treated mice week 6 post CAR infusion to identify potential tumour burden with further sectioning showing a lack of CD4 or CD8 T cells in sections cut from tumour bearing mice. Scale bars are: top panel, 1000 μm; middle panel, 500 μm; bottom panel, 200 μm.
